# Exercise postconditioning reduces ischemic injury via suppression of cerebral gluconeogenesis in rats

**DOI:** 10.1002/brb3.2805

**Published:** 2022-11-30

**Authors:** Fengwu Li, Xiaokun Geng, Roxanne Ilagan, Shangying Bai, Yuhua Chen, Yuchuan Ding

**Affiliations:** ^1^ China‐America Institute of Neuroscience Beijing Luhe Hospital, Capital Medical University Beijing China; ^2^ Department of Developmental Cell Biology Key Laboratory of Cell Biology, Ministry of Public Health, and Key Laboratory of Medical Cell Biology, Ministry of Education, China Medical University Shenyang China; ^3^ Department of Neurology Beijing Luhe Hospital, Capital Medical University Beijing China; ^4^ Department of Neurosurgery Wayne State University School of Medicine Detroit Michigan USA

**Keywords:** conditioning, gluconeogenesis, neuroprotection, PI3K/AKT/FoxO1, rehabilitation

## Abstract

Pre‐stroke exercise conditioning reduces neurovascular injury and improves functional outcomes after stroke. The goal of this study was to explore if post‐stroke exercise conditioning (PostE) reduced brain injury and whether it was associated with the regulation of gluconeogenesis. Adult rats received 2 h of middle cerebral artery (MCA) occlusion, followed by 24 h of reperfusion. Treadmill activity was then initiated 24 h after reperfusion for PostE. The severity of the brain damage was determined by infarct volume, apoptotic cell death, and neurological deficit at one and three days after reperfusion. We measured gluconeogenesis including oxaloacetate (OAA), phosphoenolpyruvate (PEP), pyruvic acid, lactate, ROS, and glucose via ELISA, as well as the location and expression of the key enzyme phosphoenolpyruvate carboxykinase (PCK)‐1/2 via immunofluorescence. We also determined upstream pathways including forkhead transcription factor (FoxO1), p‐FoxO1, 3‐kinase (PI3K)/Akt, and p‐PI3K/Akt via Western blot. Additionally, the cytoplasmic expression of p‐FoxO1 was detected by immunofluorescence. Compared to non‐exercise control, PostE (*p < .05) decreased brain infarct volumes, neurological deficits, and cell death at one and three days. PostE groups (*p < .05) saw increases in OAA and decreases in PEP, pyruvic acid, lactate, ROS, glucose levels, and tissue PCKs expression on both days. PCK‐1/2 expressions were also significantly (*p < .05) suppressed by the exercise setting. Additionally, phosphorylated PI3K, AKT, and FoxO1 protein expression were significantly induced by PostE at one and three days (*p < .05). In this study, PostE reduced brain injury after stroke, in association with activated PI3K/AKT/FoxO1 signaling, and inhibited gluconeogenesis. These results suggest the involvement of FoxO1 regulation of gluconeogenesis underlying post‐stroke neuroprotection.

## Graphical abstract

In this study, PostE reduced brain injury after stroke, in association with activated PI3K/AKT/FoxO1 signals and inhibited gluconeogenesis. These results suggest the involvement of FoxO1 regulation of gluconeogenesis underlying post–stroke neuroprotection.

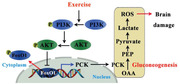

## 1 INTRODUCTION

Stroke is a leading cause of mortality worldwide (Mohsin Alvi et al., 2020). Even after survival, many individuals require costly medical care and suffer from disabilities for the remainder of their lifetimes (Wolf & Ergul, 2021). Different techniques have been studied to potentially mitigate these morbidities (Wu & Prentice, 2021). Conditioning strategies are reported to play a key role in reducing injury as well as improving functional outcomes in stroke patients (Basalay et al., 2020; Li et al., 2020). Conditioning, in the event of prolonged ischemia leading to brain infarction, is performed by introducing brief periods of ischemia and reperfusion. Conditioning before ischemia is labeled ischemia pre‐conditioning, and conditioning after is ischemia post‐conditioning. Pre‐ and post‐conditioning stimuli can also be applied in a different or remote organ, which is termed remote conditioning (Penna et al., 2015). Ischemic conditioning after cerebral ischemia, termed ischemic post‐conditioning, has been extensively studied to attenuate stroke‐induced brain tissue loss (Basalay et al., 2020). Conditioning strategies involving physical activity have also been proven beneficial (Wills & Ding, 2021). Exercise conditioning before the stroke, or exercise pre‐conditioning, has been observed to reduce brain damage and induce functional recovery post‐stroke by specifically mitigating numerous adverse outcomes such as memory, neurological, and motor function impairment (Rezaei et al., 2018). Alternatively, exercise post conditioning (PostE) has been established to improve cardiovascular function in a myocardial ischemia model (Szabo et al., 2019). With the exception of a recent study indicating its beneficial role in ischemic injury (Li et al., 2021), PostE has not been thoroughly studied in the setting of neuroprotection, and its underlying mechanisms have not been determined.

Studies suggest that cerebral ischemic injury is correlated to impaired glucose metabolism (Ji et al., 2019; Yip et al., 2016). Gluconeogenesis, one of several biochemical mechanisms which regulate blood glucose levels when stores deplete, is important for peripheral metabolism (Liu et al., 2019), but its effects on the CNS have not been thoroughly examined. Recent studies, however, have revealed its important role in neurons (Geng et al., 2021; Yip et al., 2016). Phosphoenolpyruvate carboxykinase (PCK), a key enzyme of gluconeogenesis, is reported to play a role in mediating glycolysis and aerobic oxidation, termed metabolic reprogramming (Bian et al., 2017). There are two PCK isoforms, cytoplasmic PCK‐1 and mitochondrial PCK‐2, and each catalyze the key irreversible conversion of oxaloacetate (OAA) into phosphoenolpyruvate (PEP) (Liu et al., 2019). Due to ischemia, mitochondrial oxidative phosphorylation is disrupted and glycolysis fails to generate enough energy to sustain normal brain activity. Gluconeogenesis might then be induced in an attempt to meet ATP needs (Geng et al., 2021). Because of insufficient ATP, however, gluconeogenesis may then function incorrectly leading to lactic acid accumulation and oxidative damage (Yip et al., 2016). Whether this stunted gluconeogenesis may underlie the foundation of brain damage after ischemic stroke remains to be determined.

A potential mechanism involves the forkhead transcription factor (FoxO1). It has been suggested that FoxO1 suppresses gluconeogenesis in the setting of ischemic stroke after exercise post‐conditioning. FoxO1 has been demonstrated to mediate gluconeogenesis‐related gene expression on the transcriptional level and promote glucose production (Cui et al., 2019; Liu et al., 2019; Puigserver et al., 2003). During cerebral ischemia, the mechanisms of neuron death are associated with the activation of FoxO1 and the reduction of its inhibition by phosphorylation, which causes nuclear translocation as well as regulation of downstream signals (Lehtinen et al., 2006). It has been reported that PI3K/Akt signals promote the activation of FoxO1 during brain ischemic reperfusion injury (Li et al., 2021).

Studies have demonstrated that exercise promotes synaptic plasticity by upregulating the expression of FoxO1 or the PI3K/AKT molecular pathway (Cui et al., 2019; Wang et al., 2019). Knowing this, the goal of our experiments was to examine the possible neuroprotective effects of PostE as well as its possible regulation of PI3K/AKT/FoxO1 signaling.

## 2 MATERIALS AND METHODS

Our experiments were institutionally approved by the Animal Ethics Committee of Capital Medical University (Beijing, China), in accordance with the NIH Guide for the Care and Use of Laboratory Animals. Adult Sprague‐Dawley rats (a total of 96 male rats weighing 280–300 g, Vital River Laboratory Animal Technology Co., Ltd., Beijing, China) were assigned into three groups randomly: (1) Control without middle cerebral artery occlusion (MCAO) (*n* = 12), (2) MCAO without PostE (*n* = 42), and (3) MCAO with PostE (*n* = 42). Rats were subjected to PostE 24 h after reperfusion and sacrificed at one and three days after PostE for morphological and molecular analysis. Only ipsilateral ischemic hemispheres were used for Western blot and ELISA assays.

### 2.1 Focal cerebral ischemia and reperfusion

Ischemic animal groups were subjected to 2 h of right MCAO as previously described (Li et al., 2017). Briefly, rats were anesthetized with 3% isoflurane and anesthesia was maintained with 1% isoflurane from a calibrated precision vaporizer. A 4‐0 poly‐L‐lysine‐coated nylon suture was used to occlude the middle cerebral artery through the right common carotid artery into the right internal carotid artery. Cerebral blood flow, pCO_2_, pO_2_, and body temperature were monitored continuously during surgery.

### 2.2 PostE program

After MCAO, all rats were randomly divided into post‐stroke exercise or non‐exercise groups. As previously described by us (Li et al., 2021), rats were allowed to run on a treadmill device (ZS‐PT‐II, ZS Dichuang Instruments, Inc., Beijing, China) with a moderate speed of 12 meters/minute. Each rat was subjected to treadmill exercise for 30 min every day. During the exercise period, three rats were housed together in a standard cage for the same time.

### 2.3 Cerebral infarct volume and neurological deficit

One or three days after PostE, infarct volume and neurological deficit were evaluated. As we described previously (Geng et al., 2015), the rats’ brains were cut into 2 mm thick slices and stained with 2, 3, 5‐triphenyltetrazole sodium chloride (TTC, Sigma, USA). The infarct area was determined by calculating the percentage of infarct volume relative to the total ipsilateral brain area. Twenty four hours after reperfusion, neurological deficits were evaluated to examine the severity of MCAO by using modified scoring systems (Longa and Belayev scores). The rats with scores below 1 (about 10%) were excluded from the following study.

### 2.4 TUNEL

One or three days after PostE, cell death was evaluated by using the TUNEL assay as we previously described (Li et al., 2017). Briefly, rats were anesthetized, perfused with saline, and brain‐frozen sections were prepared with 4% paraformaldehyde. The commercial TUNEL detection kit (Roche, Indianapolis, USA) was then used to determine DNA fragmentation according to the manufacturer under a fluorescence microscope (DM4000, Leica). The TUNEL index was determined by counting the positively stained cells in all cells.

### 2.5 ELISA

Commercial enzyme immunoassays were prepared, as previously described by us (Geng et al., 2015), and used to detect OAA, PEP, pyruvic acid, lactate, ROS, and glucose (MLBIO, Shanghai, China) under the microplate reader (Thermo Scientific Multiskan MK3).

### 2.6 Immunofluorescence

As previously described (Geng et al., 2021), the frozen rat brain sections were prepared and the ischemic MCA penumbra region was observed. For immunofluorescence, brain sections were incubated with a mixture of PCK1 (Proteintech Cat# 16754‐1‐AP, RRID:AB_2160031), PCK2 (Proteintech Cat# 14892‐1‐AP, RRID:AB_2160044) or p‐FoxO1 (1:100, CST, USA) and neuronal specific nuclear protein (NeuN) (Abcam Cat# ab104224, RRID:AB_10711040), followed by a mixture of Alexa Fluor 488 or 647 fluorescence tagged secondary antibodies (Abcam Cat# ab150113, RRID:AB_2576208;Abcam Cat# ab150083, RRID:AB_2714032). The images were observed by using a fluorescence microscope and a Leica TCS‐SP5 confocal microscope.

### 2.7 Protein expression

Samples from rats sacrificed one or three days after exercise were analyzed by Western blot as we previously described (Geng et al., 2015). Briefly, proteins in the ischemic brain (cortex and striatum) were extracted and probed with a primary antibody PCK‐1 (Proteintech Cat# 16754‐1‐AP, RRID:AB_2160031), PCK‐2 (Proteintech Cat# 14892‐1‐AP, RRID:AB_2160044), FoxO1 (1:1000, CST), p‐FoxO1 (1:1000), p‐AKT (Cell Signaling Technology Cat# 4060, RRID:AB_2315049), and p‐PI3K (1:1000, CST) overnight at 4°C. IgG‐HRP secondary antibodies (1:1000, Santa Cruz) for all primary antibodies were further incubated for 1 h at room temperature. A monoclonal antibody against β‐actin (1:1000, Abcam) was used as a control in protein gels. ImageJ (ImageJ 1.42, National Institutes of Health) was used to quantify protein expressions for relative grayscale.

### 2.8 Statistical analysis

The data from our study were analyzed using SPSS (SPSS Inc., Chicago, IL, USA). Differences among multiple groups were assessed using a one‐way analysis of variance (ANOVA). Post hoc comparison was detected using the least significant difference test with the significance level at *p* < .05.

## 3 RESULTS

### 3.1 Brain infarction and neurological defects

TTC images demonstrated an obvious brain infarct volume in ischemia/reperfusion rats (41.9% at 1 day, 43.1% at 3 days, *n* = 9, Table 1 and Figure S1). Infarction was significantly decreased by PostE (23.4% at 1 day, 25.4% at 3 days, *p < .05). 5‐ and 12‐point score systems saw neurological deficits in the ischemia/reperfusion group (3.33 or 7.05 at 1 day, 3.17 or 6.72 at 3 days, *n* = 9, Table 1). PostE decreased the 5‐point score (2.57 at 1 day, **p < .01; 2.22 at 3 days, *p < .05) and 12‐point score (6.30 at 1 day, *p < .05; 5.50 at 3 days, *p < .05) significantly.

**TABLE 1:** Brain infarct and neurological deficits. Infarction was significantly decreased by PostE (*p < .05 at 1 and 3 days). PostE also decreased the 5‐point score (**p < .01 at 1 day, *p < .05 at 3 days) and 12‐point score (*p < .05 at 1 and 3 days). * and ** represent PostE versus stroke

	Group	Infarct volumes	5‐points	12‐points
1 day	Stroke	41.9%	3.33 ± 0.21	7.05 ± 0.17
	PostE	23.4%*	2.57 ± 0.20**	6.30 ± 0.26*
3 days	Stroke	43.1%	3.17 ± 0.17	6.72 ± 0.22
	PostE	25.4%*	2.22 ± 0.22*	5.50 ± 0.22*

### 3.2 TUNEL

**FIGURE 1:**
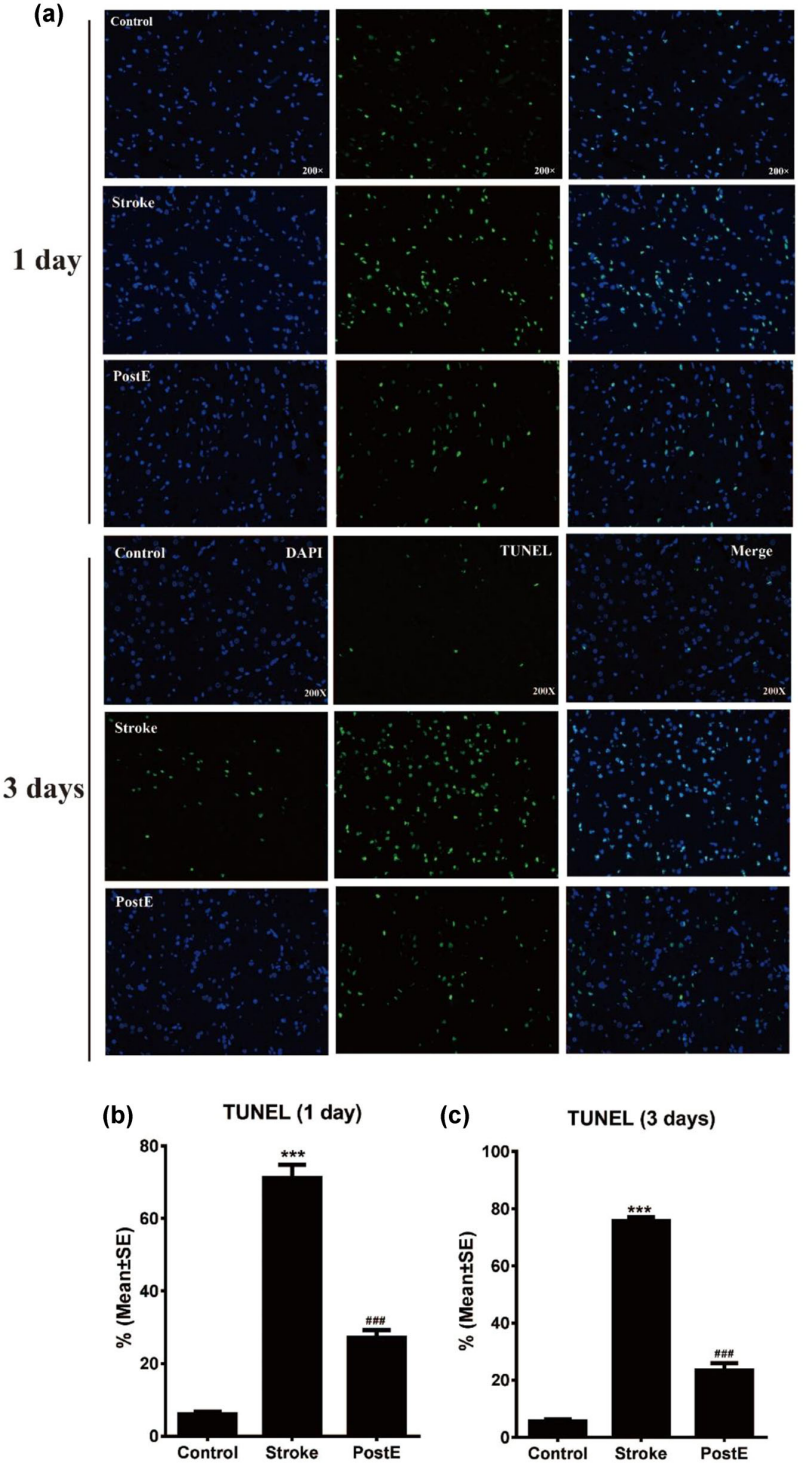
Cell death. (a) TUNEL images of PostE at 1 and 3 days after stroke. (b) Compared to the stroke group, cell death was significantly decreased after PostE protocols on days 1 and 3, respectively (^###^ *p* < .001, *n* = 6, respectively). *** represent control versus stroke; ^###^ represent PostE versus stroke

Ischemia/reperfusion‐induced significant (***p < .001) apoptotic cell death in the ischemic brain (71.7% or 75.7% at 1 or 3 days) as compared to control rats (6.0% or 5.6% at 1 or 3 days, *n* = 6, Figure 1). Cell death was significantly (^###^ *p* < .001) decreased by PostE to 27.1% at 1 day or 23.4% at 3 days (Figure 1).

### 3.3 Gluconeogenesis

**FIGURE 2:**
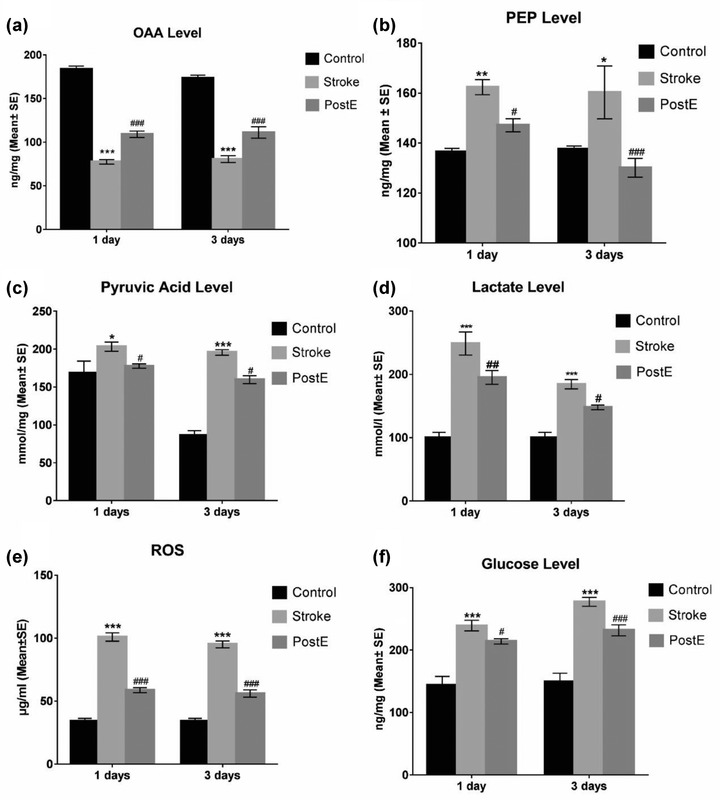
Gluconeogenesis and glucose levels. Compared to the MCAO‐only rats, PostE significantly increased levels of (a) OAA (***p < .001, at 1 and 3 days) and decreased levels of (b) PEP (*p < .05 at 1 day and ***p < .001 at 3 days), (c) pyruvic acid (*p < .05 at 1 and 3 days), (d) lactate (*p < .05 at 1 and 3 days), (e) ROS (*p < .05 at 1 and 3 days) and (f) glucose (**p < .01 at 3 days). *, ** or *** represent control versus stroke; ^#^, ^##^, or ^###^ represent PostE versus stroke

At one and three days, OAA, PEP, pyruvic acid, lactate, ROS, and glucose levels were detected (*n* = 8, Figure 2). Ischemia/reperfusion showed an obvious reduction of OAA and an increase in PEP, pyruvic acid, lactate, ROS, and glucose levels in comparison with the control rats (***p < .001). When compared to the stroke group (MCAO without exercise), PostE reversed the decreased OAA level (***p < .001 at 1 and 3 days, Figure 2a) and additionally reversed the increased PEP (*p < .05 at 1 day, ***p < .001 at 3 days, Figure 2b), pyruvic acid (*p < .05 at 1 and 3 days, Figure 2c), lactate (*p < .05 at 1 and 3 days, Figure 2d), ROS (*p < .05 at 1 and 3 days, Figure 2e) as well as glucose levels (**p < .01 at 3 days, Figure 2f). These results suggest that PostE suppressed ischemia‐induced gluconeogenesis.

### 3.4 Expression of PCKs

**FIGURE 3:**
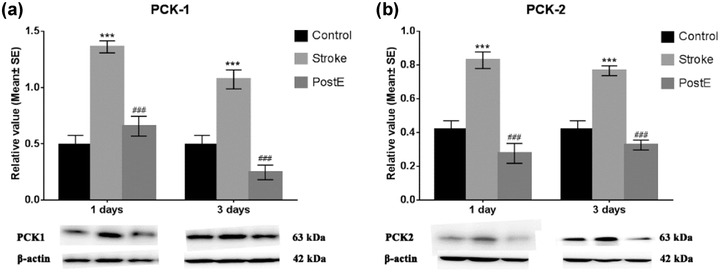
Expression of PCKs. Compared to the MCAO‐only rats, PostE reduced protein expressions of (a) PCK‐1 (***p < .001 at 1 day and *p < .05 at 3 days) and (b) PCK‐2 (***p < .001 at 1 and 3 days). * and *** represent PostE versus stroke

**FIGURE 4:**
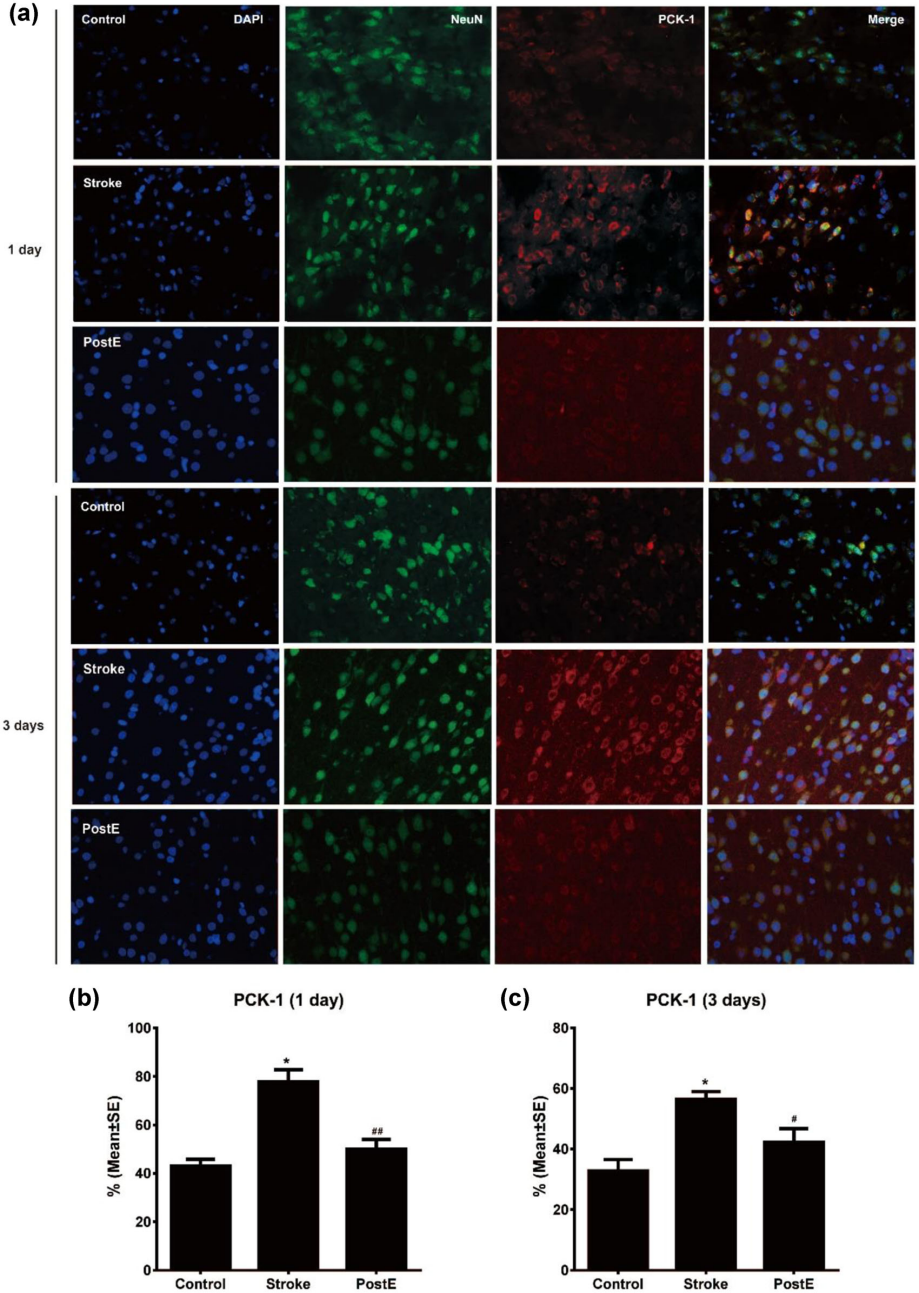
Expression of PCK‐1 on neurons. (a) Fluorescence images of PCK‐1 on neurons. (b,c) Quantification of the fluorescence images show the increasing expression of PCK‐1 in neurons after stroke (*n* = 6, respectively, PCK‐1, *p < .05 on both days). In comparison to the stroke group, exercise post‐conditioning significantly reduced PCK‐1 expressions (^##^ *p* < .01 at 1 day, ^#^ *p* < .05 at 3 days). * represents control versus stroke; ^#^ and ^##^ represent PostE versus stroke

**FIGURE 5:**
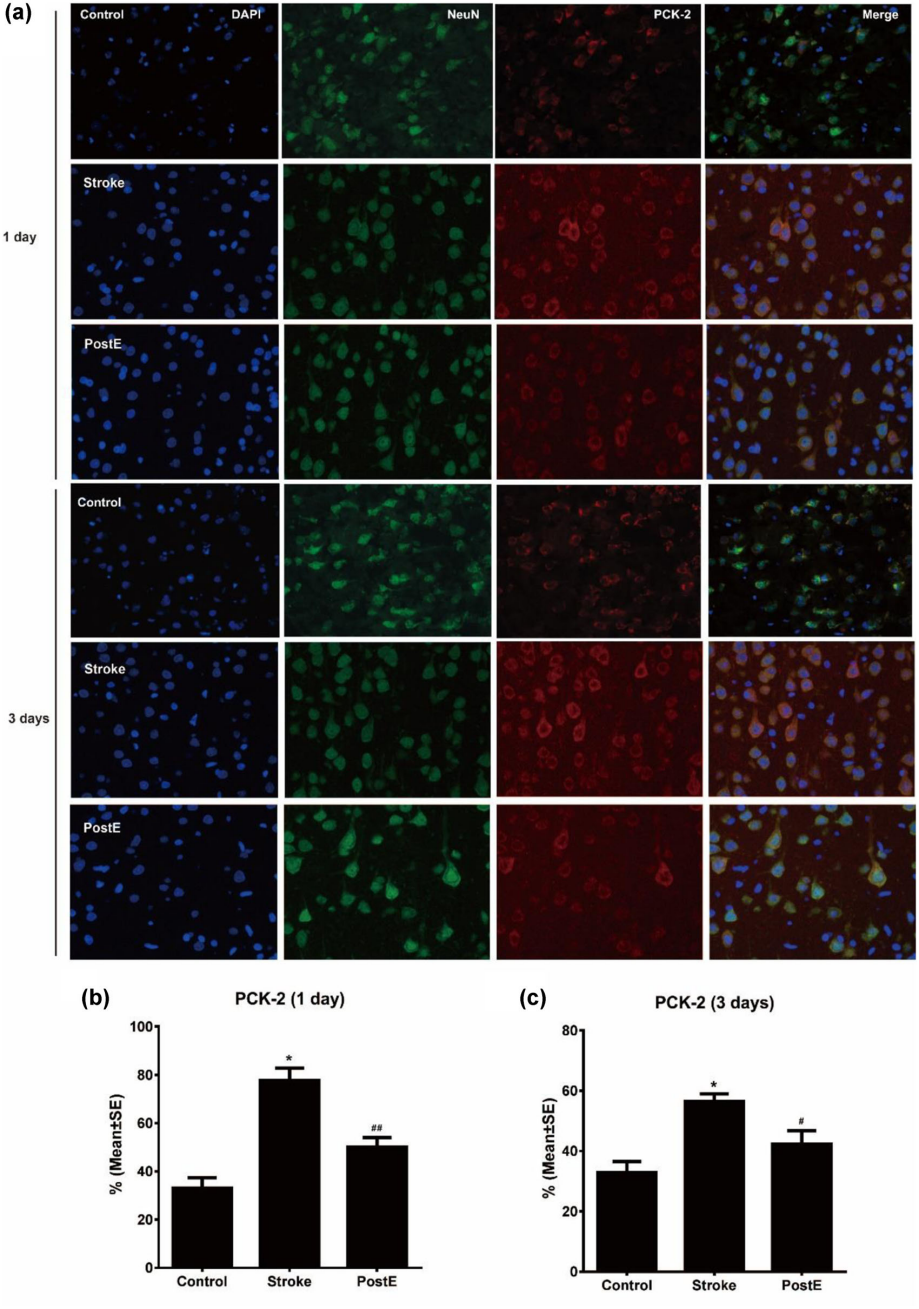
Expression of PCK‐2 on neurons. (a) Fluorescence images of PCK‐2 on neurons. (b,c) Quantification of the fluorescence images show the increasing expression of PCK‐2 in neurons after stroke (*n* = 6 respectively, PCK‐2, *p < .05 at 1 and 3 days). In comparison to the stroke group, exercise post‐conditioning significantly decreased PCK‐2 expressions (^##^ *p* < .01 at 1 day and ^#^ *p* < .05 at 3 days). * represents control versus stroke; ^#^ and ^##^ represent PostE versus stroke

Ischemia/reperfusion significantly (***p < .001) enhanced the protein expression of PCK‐1 and PCK‐2. PostE largely reduced the high expressions of PCK‐1 (***p < .001 at 1 day, *p < .05 at 3 days, Figure 3a) as well as PCK‐2 (***p < .001 at 1 and 3 days, Figure 3b) after stroke. The confocal images with double immunofluorescence labeling show the same expression trends of PCKs in neurons. Neuronal expression of both PCK1 and 2 were upregulated after stroke (*n* = 6 respectively, PCK‐1, *p < .05 at 1 and 3 days, Figure 4; PCK‐2, *p < .05 at 1 and 3 days, Figure 5). PostE decreased the cellular expressions of PCK‐1 (^##^ *p* < .01 at 1 day and ^#^ *p* < .05 at 3 days, Figure 4) and PCK‐2 (^##^ *p* < .01, at 1 day and ^#^ *p* < .05 at 3 days, Figure 5). These results suggest that PostE reduced the expression of gluconeogenetic key enzymes, particularly in neurons after stroke.

### 3.5 Phosphorylation of PI3K/AKT/FoxO1 and total

**FIGURE 6:**
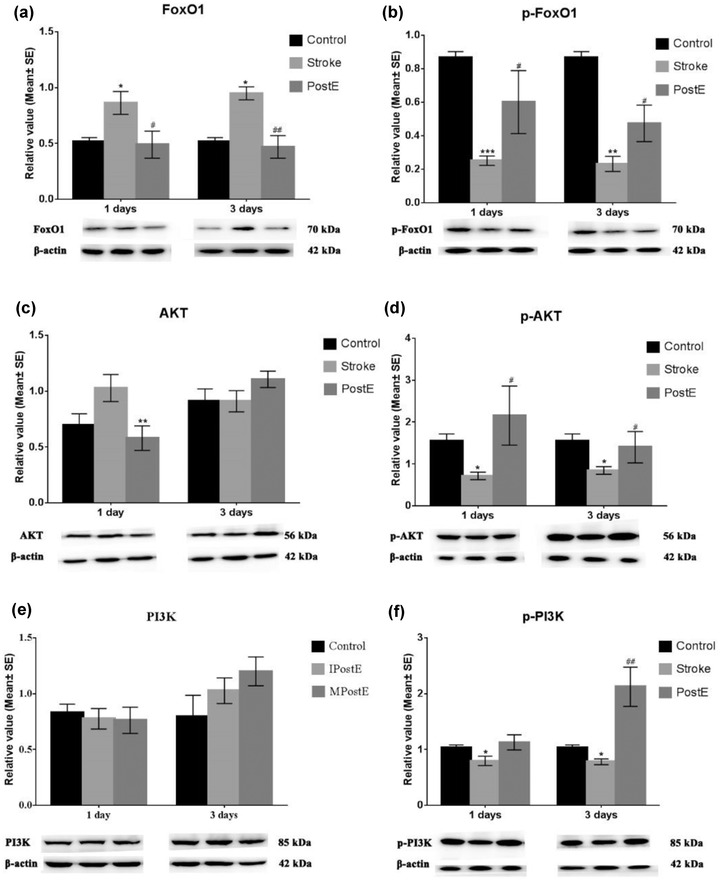
Expression of PI3K/Akt/FoxO1 signals. Alteration of (a) FoxO1, (b) p‐FoxO1, (c) AKT, (d) p‐AKT, (e) PI3K, and (f) p‐PI3K protein expression after PostE were demonstrated. In comparison to the stroke group, PostE reduced expressions of (a) FoxO1 (*p < .05, at 1 and 3 days) and (c) AKT (**p < .01, at 1 day). PostE further significantly increased expressions of (b) p‐FoxO1 (*p < .05, at 1 and 3 days), (d) p‐AKT (*p < .05, at 1 and 3 days), (f) p‐PI3K (*p < .05, at 3 days). *, **, or *** represent control versus stroke; ^#^, ^##^, or ^###^ represent PostE versus stroke

Compared to control rats, an obvious increase (*p < .05) in total FoxO1 and a decrease in phosphorylation of PI3K/ AKT/FoxO1 was discovered in the MCAO rats (Figure 6). Again, PostE reversed the expressions of FoxO1 (*p < .05, at 1 and 3 days, Figure 6a) and AKT (**p < .01, at 1 day, Figure 6c). In addition, PostE increased expressions of p‐FoxO1 (*p < .05, at 1 and 3 days, Figure 6b), p‐AKT (*p < .05, at 1 and 3 days, Figure 6d), and p‐PI3K (*p < .05, at 3 days, Figure 6f). The data suggest that PostE decreased post‐stroke gluconeogenesis by activating phosphorylation of PI3K/AKT/FoxO1 signaling.

### 3.6 Cytoplasmic expression of p‐FoxO1

**FIGURE 7:**
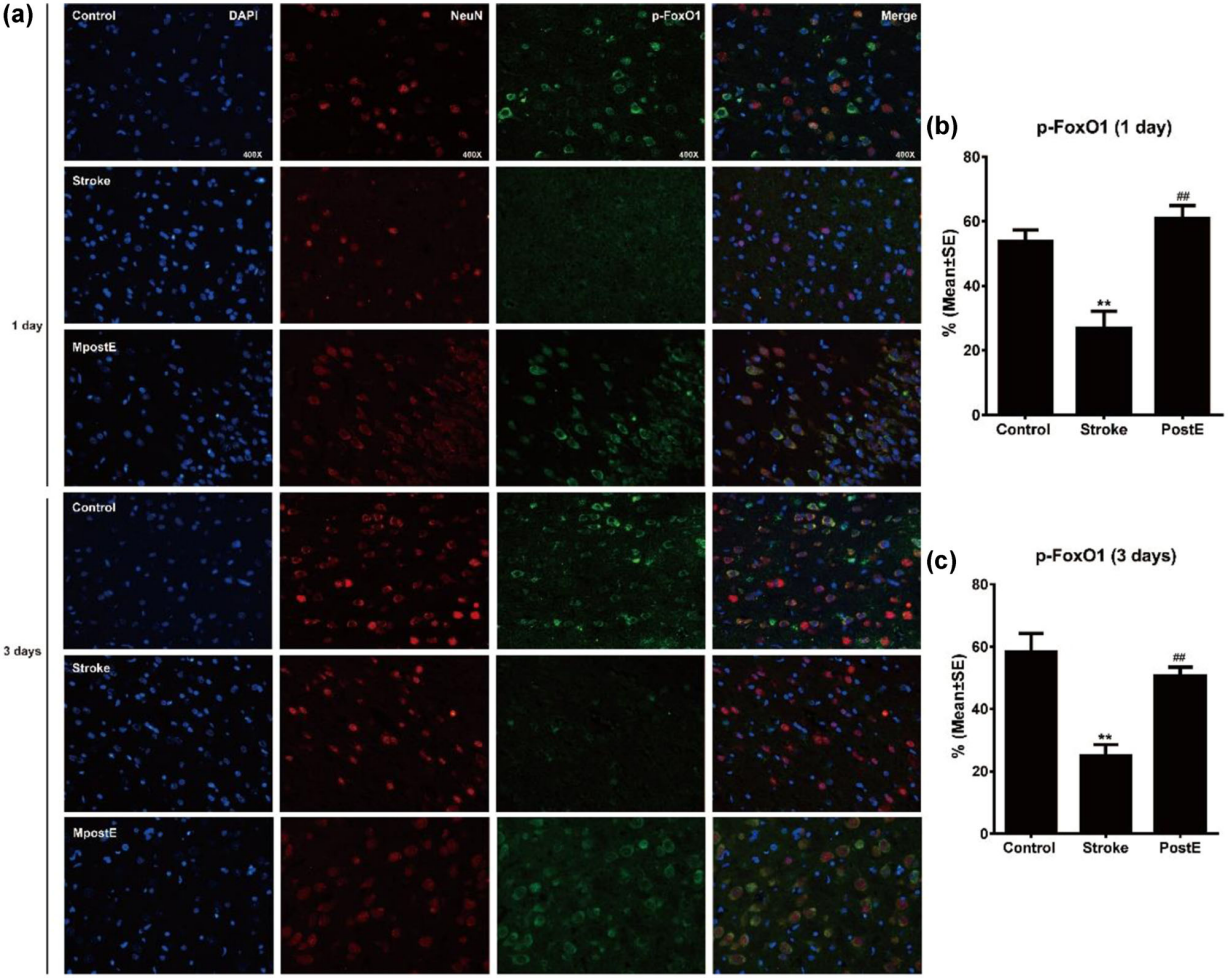
Cytoplasmic expression of p‐FoxO1. (a) Fluorescence images of cytoplasmic p‐FoxO1 expression in neurons. (b,c) Quantification of the fluorescence images demonstrate increasing cytoplasmic expression of p‐FoxO1 in neurons after PostE in comparison to the stroke group (*n* = 6 respectively, **p < .01 on both days). ** represents control versus stroke; ^##^ represent PostE versus stroke

The fluorescence images illustrate the cytoplasmic expression of p‐FoxO1 in neurons (Figure 7). Compared to control rats, ischemia/reperfusion significantly reduced cytoplasmic expression of p‐FoxO1 (**p < .01). However, PostE reversed the reduction in cytoplasmic expression of p‐FoxO1 (*n* = 6 respectively, ^**^ *p* < .01 at 1 day and 3 days, Figure 7), suggesting that PostE stimulates FoxO1 cytoplasmic expression.

## 4 DISCUSSION

**FIGURE 8:**
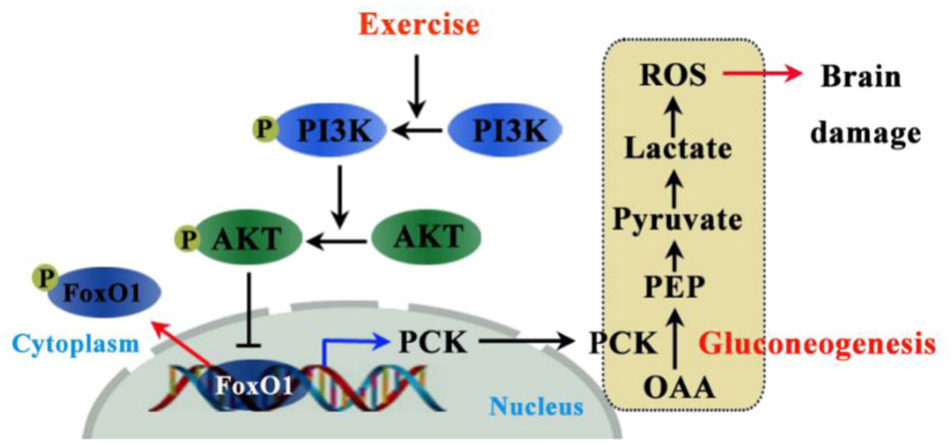
A schematic illustration of the proposed mechanism for exercise post‐conditioning reducing brain injury by restraining gluconeogenesis signals. Exercise induces phosphorylation of PI3K/AKT/FoxO1 signals, which induces FoxO1 translocation to the cytoplasm, deactivating its gene transcription function. This deactivated gene transcription decreases the expression of PCKs

In this study, exercise post‐conditioning exhibited a neuroprotective effect by reducing brain damage after an ischemic stroke. Exercise post‐conditioning reduces brain infarction and apoptotic cell death, as well as alleviates neurological deficits at one and three days. Decreased brain damage was accompanied by a decreased level of gluconeogenesis in neurons and activation of PI3K/AKT/FoxO1 signals (Figure 8). Although the present study did not address their cause‐and‐effect relationship, as the first step in this topic, we found an association between PI3K/AKT/FoxO1 signals and gluconeogenesis following ischemia and reperfusion injury.

The mechanisms of ischemic and exercise conditioning are probably based on protective cellular pathways, such as suppression of oxidative stress, autophagy or apoptosis, and the inflammatory response (Dornbos & Ding, 2012; Gao et al., 2021; Li et al., 2017; Zhang et al., 2019; W. Zhao et al., 2018). Pre‐ischemic exercise has been found to exert neuroprotective effects by alleviating brain damage and improving functional outcomes (Sakakima, 2019). Our data also indicates that post‐stroke exercise conditioning has the ability to produce beneficial brain protection effects, just like exercise preconditioning (F. Li et al., 2017). The post‐ischemic exercise was initially reported to exert beneficial post‐conditioning effects via ameliorating antioxidative statuses in myocardial infarction (Szabo et al., 2019); they suggested that exercise post‐conditioning following intervention inhibited post‐ischemia myocardial damage (Szabo et al., 2019). In addition, post‐stroke exercise also contributed to neuroplasticity and functional recovery by inducing brain‐derived neurotrophic factor (BDNF) secretion and suppressing endoplasmic reticulum stress (F. Li et al., 2021).

Ischemic injury was reported to relate to impaired glucose metabolism (Robbins & Swanson, 2014). During ischemia, when mitochondrial oxidative phosphorylation and ATP production are disrupted, anaerobic glycolysis becomes the primary source of ATP. Anaerobic glycolysis alone cannot produce sufficient ATP to maintain brain functioning, instead, lactate production leads to acidosis and ROS (Geng et al., 2017). When reperfusion occurs, mitochondrial dysfunction contributes to a lower ATP level. It is expected that gluconeogenesis would increase after ischemia to provide additional substrate for energy production (Geng et al., 2017; Geng et al., 2021). Normally, the upregulation of gluconeogenesis leads to an increase in glucose production for the generation of ATP (Han et al., 2016; Petersen et al., 2017). However, as an ATP‐dependent process, gluconeogenesis can further deplete ATP stores (Kim et al., 2019; Yip et al., 2016). This stunted gluconeogenesis will lead to excess PEP activity, in turn augmenting lactic acidosis (Rabinowitz & Enerback, 2020), and ROS production. These processes are well established to cause cell death, (Geng et al., 2021; K. C. Wu et al., 2017) since the formation of glucose will be interrupted by lack of ATP.

PCK acts as a vital enzyme for glucose metabolism by catalyzing gluconeogenesis’ key irreversible process—the decarboxylation and following phosphorylation of OAA to generate PEP (Chung et al., 2015). A previous study demonstrated that exercise training reduces hepatic pyruvate carboxylase and gluconeogenesis (Pereira et al., 2020), suggesting a potential neuroprotective effect from exercise post‐conditioning. In the present study, a large increase in OAA as well as a large decrease in PEP and glucose was found after exercise post‐conditioning. These findings are in accordance with prior results which demonstrated that post‐stroke hypothermia interventions decreased glucose levels toward sham levels and mediated OAA and PEP (F. Li et al., 2021). Additionally, we saw a significant decrease in PCK1/2 expression with exercise post‐conditioning. This rate‐limiting enzyme of gluconeogenesis is also demonstrated to decrease in the liver after exercise training (Souza Pauli et al., 2014). These findings suggest that exercise post‐conditioning potentially suppresses gluconeogenesis to reduce neural damage.

This study indicated that gluconeogenesis occurred in neurons with the expression of PCKs (PCK‐1 and PCK‐2). These results were consistent with our prior study, which demonstrated the presence of PCKs in glia and neurons, and found that PCK expression was increased after ischemic stroke (Geng et al., 2021). Although the underlying mechanism driving the switch from glycolysis to gluconeogenesis remains unknown, it is believed that phosphofructokinase activation may participate in this process (Yip et al., 2016).

FoxO1 belongs to the forkhead family of transcriptional regulators which exerts pro‐apoptotic effects on neurons when activated (K. J. Wang et al., 2020). Activation and nuclear translocation of FoxO1 cause itself and its target genes to translocate from the cytoplasm to the nucleus (Sakaguchi et al., 2019). Phosphorylation deactivates FoxO1 and induces translocation into the cytoplasm (Sakaguchi et al., 2019). Studies have demonstrated that FoxO1 regulates hepatic gluconeogenesis (K. Zhang et al., 2019). FoxO1 binds the promotor of and increases gene expression of PCK1, the key rate‑limiting gluconeogenic enzymes, and thus upregulates hepatic glucose (P. Zhang et al., 2014). Studies in hepatoma cells suggest that FoxO1 controls the transcription of reporter genes containing the PCK promoters (Cao et al., 2018). FoxO1 transcriptionally regulates PCK in the pyruvate‑PEP futile cycle (Puigserver et al., 2003). In agreement with previous results (Ouyang et al., 2012), we saw a higher level of FoxO1 expression in the cytoplasm compared to the nucleus after exercise post‐conditioning, suggesting that this exercise intervention deactivated FoxO1 after stroke. Studies have demonstrated that FoxO1‐mediated apoptosis induces apoptotic factors including Fas, TNF‐α, and Bim via a consensus FoxO1 binding site (C. H. Wu et al., 2019; X. Zhang et al., 2018). Additionally, activation or phosphorylation of FoxO1 is shown to induce hepatic gluconeogenesis (Puigserver et al., 2003), which indicates the potential regulation of FoxO1 on neural gluconeogenesis.

It is well established that the conditioning strategy protects neuronal injury against ischemia by activation of the FoxO signaling pathway (Zhan et al., 2010). Ischemic pre‐ or post‐conditioning was reported to induce neuroprotection via activation of the SIRT1/FoxO1 signaling pathway in rats with global cerebral ischemia (Liu et al., 2021; Zhan et al., 2010). Additionally, ischemic conditioning was demonstrated to suppress hepatic gluconeogenesis and enhance glucose uptake via a brain‐liver neurocircuit in diabetic mice (Kurabayashi et al., 2020; Kurabayashi et al., 2018). Renal ischemic preconditioning improved renal ischemia/reperfusion injury by decreasing gluconeogenesis and hyperglycemia (J. R. Li et al., 2019). Exercise is reported to exert its therapeutic influence on Alzheimer's disease through the targeting FoxO1(N. Zhao, Xia, & Xu, 2021) and by reducing brain damage by increasing Bcl‐2 and decreasing caspase‐3 and BAX expression in cerebral ischemia (Lin et al., 2021; Wang et al., 2019). This suggests that exercise post‐conditioning reduces neuron apoptosis through FoxO1‐meditated gluconeogenesis. A link between FoxO1 to gluconeogenesis was suggested in the present study, and further investigations are warranted to examine a cause‐and‐effect relationship.

The activation of PI3K/AKT signaling has been reported to attenuate brain damage after focal cerebral ischemia (Park et al. 2019). Studies have shown that the PI3K/AKT pathway mediates FoxO1 after brain ischemia‐reperfusion damage (He et al., 2019) and is activated by exercise (Lin et al., 2020; Wu et al., 2018). In normal settings, AKT downregulates FoxO1 signaling by phosphorylation at three phosphorylation sites: Thr24, Ser256, and Ser319 (Zhang et al., 2018). During ischemia, phosphorylation of PI3K/Akt is inhibited causing decreased FoxO1 phosphorylation as well as increased nuclear translocation (He et al., 2019; Wang et al., 2020) and gluconeogenesis regulation (Pitaloka et al., 2019). Recent studies showed that mebhydrolin or puerarin ameliorated blood glucose homeostasis by suppressing hepatic gluconeogenesis via activation of the PI3K/AKT/FoxO1 pathway in T2DM mice (Liu et al., 2021; Zhao et al., 2021). These results indicate the potential role of the PI3K/AKT/FoxO1 pathway in regulating brain gluconeogenesis. Although we did not study the regulation of these signal pathways, our present data suggested that PI3K/AKT/FoxO1 pathways may regulate gluconeogenesis. Our further study would focus on the possible regulatory mechanism. Overall, our present results indicate that exercise post‐conditioning reduces post‐stroke infarct via decreasing the PI3K/Akt axis and subsequently increasing the phosphorylated FoxO1 level. These findings were supported by previous studies that irisin reduces gluconeogenesis via PI3K/Akt/ FoxO1 mediated PCK signals (Liu et al., 2015).

In summary, we demonstrated a neuroprotective effect of PostE after stroke due to its reduction of gluconeogenesis levels and modulation of PI3K/AKT/FoxO1 signaling.

## AUTHOR CONTRIBUTIONS

Fengwu Li conducted the animal, biochemical experiments employed in this research and wrote the draft. Fengwu Li, Xiaokun Geng, Shangying Bai, Roxanne Ilagan, and Yuhua Chen were instrumental in preparing the manuscript and the language polishing. Fengwu Li, Xiaokun Geng, and Yuchuan Ding were responsible for the experimental design. All authors contributed to the article and approved the submitted version.

## CONFLICT OF INTEREST

The authors declare no competing financial interests.

### PEER REVIEW

The peer review history for this article is available at: https://publons.com/publon/10.1002/brb3.2805.

## Supporting information


**Supporting Information Figure S1**. 2,3,5‐triphenyltetrazolium chloride (TTC) histology image of infarct volume reduction by PostE.

## Data Availability

The data that support the findings of this study are available from the corresponding author upon reasonable request.
