# Supplementary figures and images for: Exercise postconditioning reduces ischemic injury via suppression of cerebral gluconeogenesis in rats
Source: Brain Behav. 2022 Nov 30;13(1):e2805. doi: 10.1002/brb3.2805 (PMC9847623; doi:10.1002/brb3.2805)

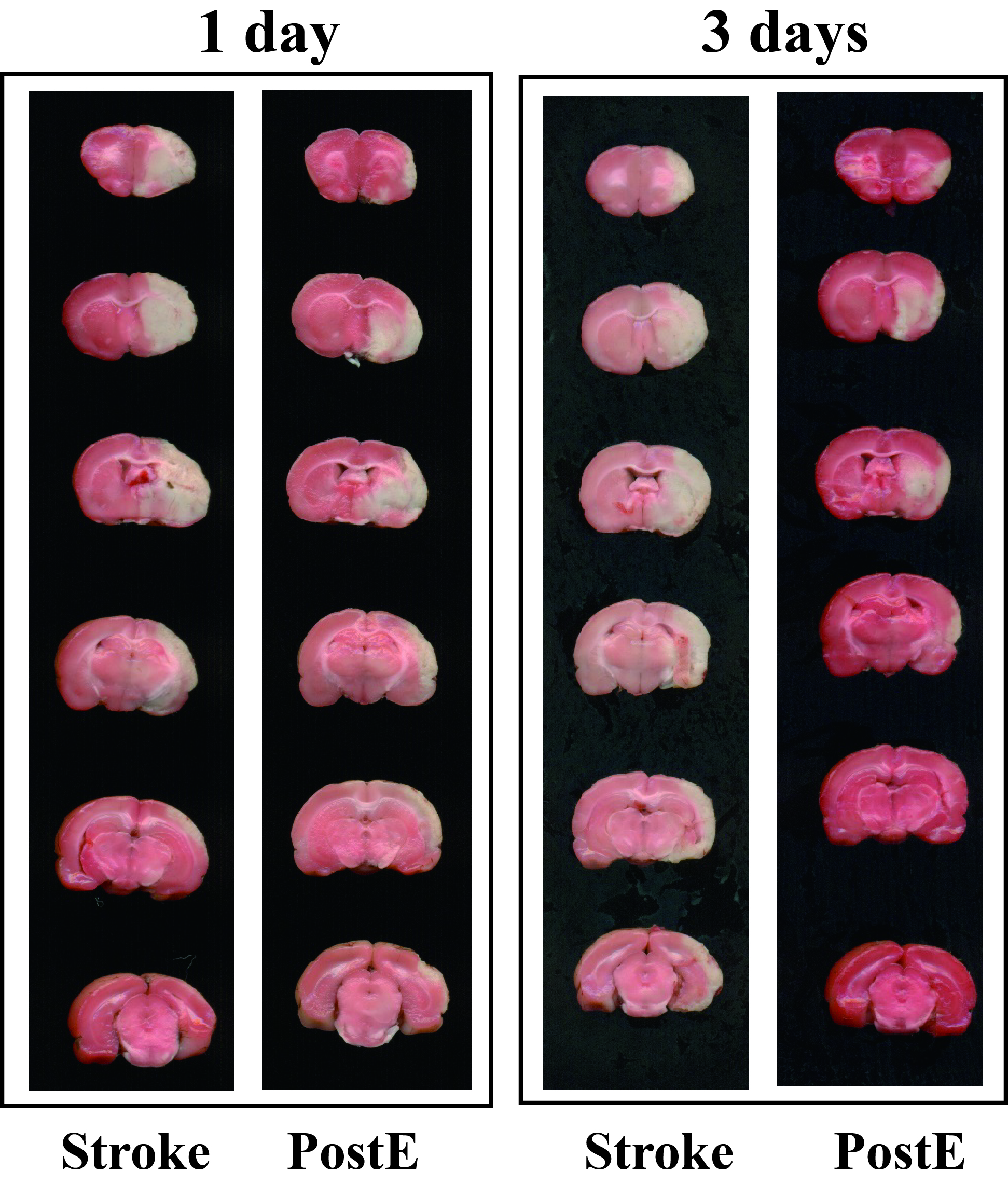

Supplement: Supplementary file 1 — Supporting Information Figure S1. 2,3,5‐triphenyltetrazolium chloride (TTC) histology image of infarct volume reduction by PostE. [file BRB3-13-e2805-s001.jpg]
